# Dermoscopy of cutaneous metastasis of colorectal adenocarcinoma in a patient with phototype V

**DOI:** 10.1016/j.jdcr.2025.11.034

**Published:** 2026-01-16

**Authors:** Esther Verónica Echeverry Rodriguez, Janeth del Pilar Villanueva-Reyes, Carolina Concha Arango, Laura Manuela Pulgarin

**Affiliations:** a Dermatology and Dermatologic Surgery Section, Universidad del Valle, Cali, Colombia; b Dermatology section, Hospital Universitario del Valle, Cali, Colombia; c Dermatology Section, Universidad de Antioquia, Medellín, Colombia

**Keywords:** dermoscopy, cutaneous metastasis, colorectal adenocarcinoma, phototype V, peppering-like pigment

A 52-year-old man presented with a 4-month history of pink pruritic papules in the pubic area. He was undergoing treatment for stage IIIB colorectal adenocarcinoma, diagnosed 2 years earlier. On physical examination, he had skin phototype V and showed pink papules coalescing into an indurated, hyperpigmented plaque on his abdomen, scrotum, and right leg (Figs 1 and 2, *A*). He also had lymphedema of the legs, penis, and scrotum.

**Fig 1:**
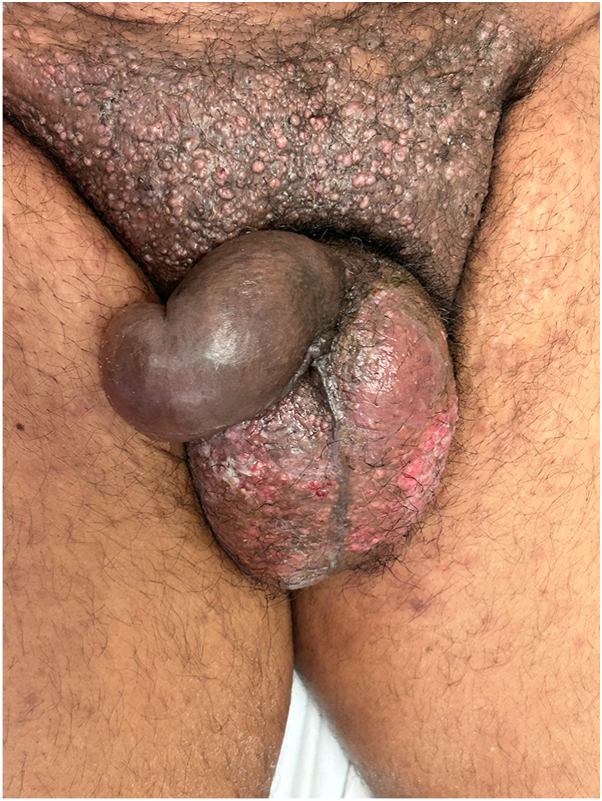
Clinical photo. Grouped papules on the pubis forming a hardened, hyperpigmented plaque with a shell-like appearance. Some lesions are excoriated.

**Fig 2:**
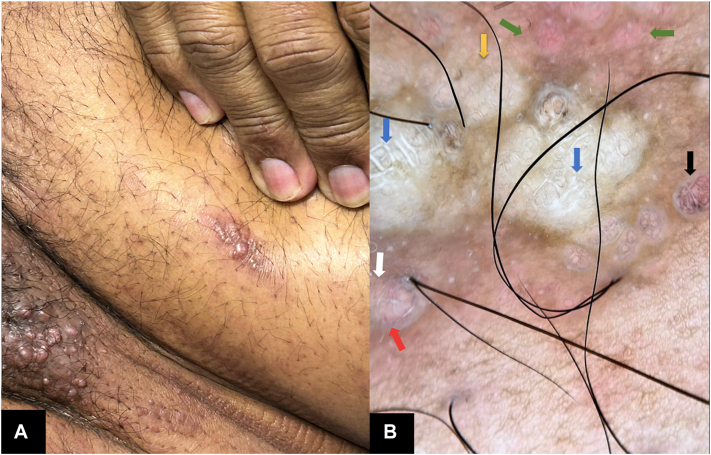
Abdomen papules. **(A)** Clinical Photo. **(B)** Dermoscopy on polarized light. Globular structures with bright white septa forming a saccular pattern (*blue arrows*), surrounded by hyperpigmentation, dotted pigment with a pepeering-like (*black arrows*), with a fine network. White areas without structure (*yellow arrow*). Pale pink globular structures (*green arrows*). Punctate vessels (*white arrow*). Short, irregular linear vessels (*red arrow*).

Dermoscopy revealed globular structures with a saccular pattern, associated with bright white septa and brown borders, on a structureless white background with peppering-like pigmentation. The lesions were surrounded by pale pink globular areas, giving them an erythematous appearance. Scattered punctate vascular structures, short linear vessels, and irregular linear vessels were also identified (Figs 2 and 3, *A*, *B*).

**Fig 3:**
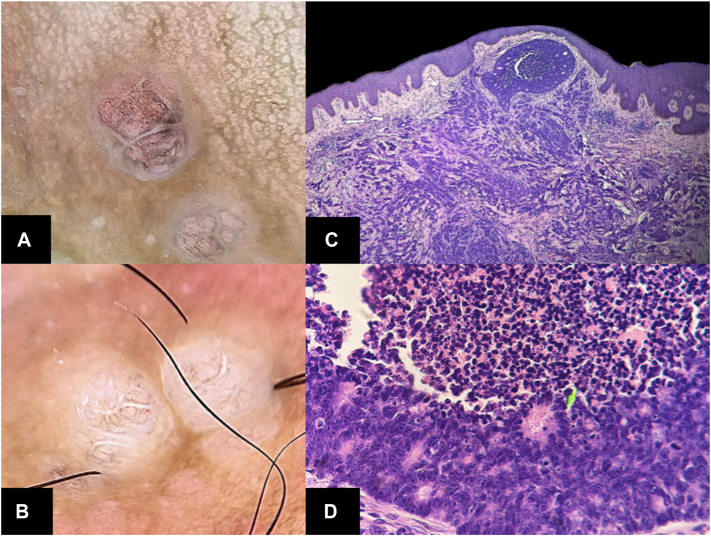
Cutaneous metastases from colorectal carcinoma**. (A)** Dermoscopy on polarized light. Close-up of globular structures with peppering-like punctate pigment. **(B)** Dermoscopy on polarized light. Areas with punctate vascular structures. **(C)** Malignant tumor infiltrating the dermis, proliferation of atypical glands, cells with irregular cytoplasm, pleomorphism, prominent nuclei, and atypical mitotic figures. Areas of central necrosis and desmoplastic stroma. **(D)** Accumulation of mucinous cells in the papillary dermis, (*green arrow*), generating a circular neoformation with central necrosis. (**C** and **D,** H&E stain; original magnifications: **C,** ×10; **D,** ×40).

Histopathological examination showed lymphatic invasion and infiltration of malignant glandular epithelial cells in the desmoplastic dermis, with morphology similar to the primary colorectal tumor (Fig 3), confirming the diagnosis of cutaneous metastasis.

Cutaneous metastases from colorectal carcinomas (CMCCs) are rare.^1^ Dermatoscopic reports of CMCC in light-skinned patients describe the main dermatoscopic findings as a polymorphic vascular pattern and a white, pinkish, or reddish background without defined structures.^2^, ^3^, ^4^, ^5^, ^6^ Since vascular patterns are more difficult to observe in dermoscopy of high-phototype patients, the description of new structures in these individuals could lead to earlier diagnoses and improved prognosis.^2^ Our patient presented saccular structures with bright white septa and peppering-like pigmentation, adding new CMCC findings. This case highlighted the need for further dermatoscopic documentation in patients with skin of color. The correlation of dermatoscopy structures is described in Table I.^2^^,^^7^^,^^8^

**Table I:** Correlation of dermoscopy structures and histopathological findings of colorectal metastasis

Dermoscopy findings	Histopathological correlation	Reference
Bright white partitions that form saccular structures	Thickened collagen bundles/desmoplastic stroma, associated with tumor infiltration in the dermis	Marghoob et al^7^ (2009) Massone et al^8^ (2021)
Structureless white area on a pink to red background	New collagen remodeling in the dermis	Chernoff, et al^2^ (2014) Marghoob et al^7^ (2009)
Vascular structures	Disorganized neo-angiogenesis	Chernoff et al^2^ (2014)
∗Saccular structures with peppering-like pigment	Melanophages or melanin deposition adjacent to tumor nests	Authors’ theory, based on assone et al^8^ (2021)

∗ Patients with dark-skin.

## Conflicts of interest

None disclosed.
